# A Strain-Based Method to Estimate Slip Angle and Tire Working Conditions for Intelligent Tires Using Fuzzy Logic

**DOI:** 10.3390/s17040874

**Published:** 2017-04-16

**Authors:** Daniel Garcia-Pozuelo, Jorge Yunta, Oluremi Olatunbosun, Xiaoguang Yang, Vicente Diaz

**Affiliations:** 1 Mechanical Engineering Department, Universidad Carlos III de Madrid, Avd. De la Universidad, Madrid 28911, Spain; dgramos@ing.uc3m.es (D.G.-P.); vdiaz@ing.uc3m.es (V.D.); 2 School of Mechanical Engineering, University of Birmingham, Edgbaston B15 2TT, UK; o.a.olatunbosun@bham.ac.uk; 3 Vanlead Rubber & Tire Research Institute, Wanli Tire Corporation Limited, Guangzhou 510425, China; neuasyang1@aliyun.com

**Keywords:** intelligent tires, strain gauges, slip angle estimation, tire conditions, fuzzy logic

## Abstract

Tires equipped with sensors, the so-called “intelligent tires”, can provide vital information for control systems, drivers and external users. In this research, tire dynamic strain characteristics in cornering conditions are collected and analysed in relation to the variation of tire working conditions, such as inflation pressure, rolling speed, vertical load and slip angle. An experimental tire strain-based prototype and an indoor tire test rig are used to demonstrate the suitability of strain sensors to establish relations between strain data and lateral force. The results of experiments show that strain values drop sharply when lateral force is decreasing, which can be used to predict tire slip conditions. As a first approach to estimate some tire working conditions, such as the slip angle and vertical load, a fuzzy logic method has been developed. The simulation and test results confirm the feasibility of strain sensors and the proposed computational model to solve the non-linearity characteristics of the tires’ parameters and turn tires into a source of useful information.

## 1. Introduction

Up to now, tires have always been passive elements which have a major impact on ride comfort, fuel consumption, and vehicle safety and stability. However, the lack of essential information regarding the characteristics of the tire-road contact is the main reason to develop an intelligent tire as a warning system for road conditions, for optimized braking and accelerating control on poor surfaces and as a tire fault detection system. The provided information may be used individually by current control systems, for instance, Electronic Stability Control (ESC), Traction Control System (TCS) or Anti-lock Braking System (ABS). In this way, an intelligent tire could help to avoid potentially hazardous situations and represents a crucial element for vehicle’s safety. The intelligent tire concept would have the final aim of monitoring, in real time, longitudinal and lateral forces at the contact patch, potential adherence, slip angle, etc.

**Figure 1:**
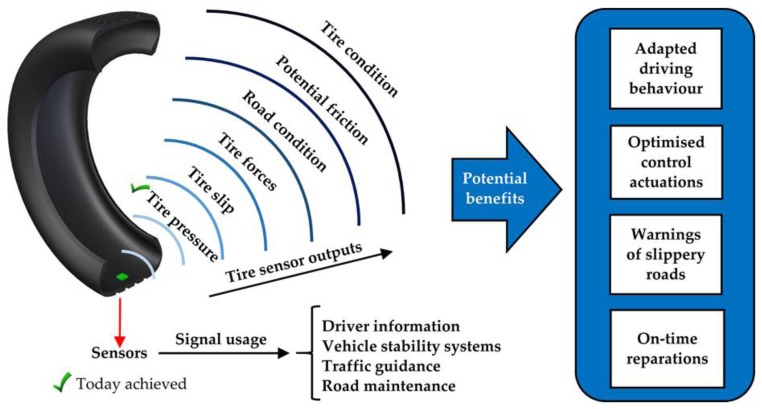
Intelligent tire sensor outputs and potential benefits of intelligent tires.

Concerning intelligent tires’ developments, in 2002, the USA passed the Tread Act for Tire Pressure Monitoring System (TPMS) to warn the driver if a tire is significantly under-inflated [1], this being the first such product introduced in the automotive market. Some years later, the EU followed the same path. Over the past several years, some research groups have also carried out intensive research into the intelligent tire concept [2], demonstrating that, although TPMS was a great milestone, the intelligent tire idea has bigger prospects than just TPMS, as shown in Figure 1.

Due to the non-linearity of the tire-road contact characteristics, it is complicated to apply the brake pedal force without locking the wheels, which may result in the loss of adherence and a traffic accident unless an ABS takes action. Moreover, this situation could be even worse when a vehicle is travelling on wet or muddy surfaces where friction is changing [3].

During the past decades, much more information has become available on the influence of tire parameters in tire tread behaviour. Overall, most of them approach tire deformation measurement, among other tire mechanical parameters by means of different sensors. Successful results concerning tread acceleration measurements and wireless tire data transmission systems were presented by Negrus et al. [4]. In [5], a magnetic sensor to measure features such as radial and circumferential deformation on the tire tread in order to find relations between deformation and forces of the tire was developed. A study by Stelzer et al. [6] demonstrated that it is possible to measure the road friction coefficient using Surface Acoustic Wave (SAW) sensors. In recent years, much of the current literature on tires’ development has focused on the estimation of the road friction coefficient [7,8,9]. A recent study by Hong et al. [10] presented an algorithm that estimates the contact patch length and the friction coefficient using the radial and lateral acceleration profiles of the tire-road contact patch, respectively. Cheli et al. developed a device capable of estimating forces at the contact patch in real time [11].

The main idea behind Tuononen’s research [12,13] was to provide estimations about the vehicle state through measuring tire carcass deflections using an optical sensor. Cullen et al. [14] also obtained successful measurements of inflation pressure and tire strain using segmented capacitance rings. Finally, Magori et al. [15] installed an ultrasonic sensor on the base of a wheel rim to monitor the contact patch deformation besides some additional tire features.

Although the information provided by these systems is useful in a variety of situations, their viability in the future is subject to the possibility of real-time implementation, which is considered as the main hindrance. To solve this, many obstacles must be taken into account, for instance, the compatibility of the sensors with the characteristics of tire rubber (mainly stiffness issues), data transmission or economic feasibility relating to the use of expensive sensors, but above all the main problem is how to meet the power requirements of all the electronic devices. In [16], Yilmazoglu et al. proposed an optimized material system that has the prospect of saving energy for sensor implementation within battery-operated measurement systems. Singh et al. [17] showed the viability of an onboard system for harvesting vibration energy from tires to supply electric components with energy.

The purpose of this paper is to demonstrate the potential and suitability of strain gauges to estimate slip angle and vertical load by means of fuzzy logic. To do this, the analysis of the influence of these parameters in combination with other tire working conditions such as inflation pressure and rolling speed on the tire tread strain behaviour has been developed.

There is a large volume of published studies describing the role of slip angle (i.e., cornering conditions) and its estimation as a crucial factor for the tire and vehicle behaviour [18,19,20]. Lateral slip conditions and the grip limit of the tire are essential information for vehicle stability control during cornering, because lateral forces demand higher grip forces at the tire-road contact patch. Phanomchoeng et al. [21] used a nonlinear vehicle model to estimate the slip angle which was validated with experimental measurements on a test vehicle and is suitable for a large range of operating conditions. A new tire–road friction coefficient estimation algorithm based on measurements related to the vehicle´s lateral dynamics as a function of slip angle (among other parameters) was developed by Hahn et al. [22]. Recently, a piezoelectric sensor to measure the sidewall deflection was used by Erdogan et al. [18], who obtained successful estimations of the slip angle up to 5°.

On the other hand, the influence of inflation pressure, speed and vertical load under cornering conditions on tire tread deformation is also analysed herein. Although all these features affect the tire tread behaviour, many studies indicate that vertical load is the most influential for potential grip as well as for tread deformation [12,13,15]. As it is well known, when the load transfer is increased, the effective grip is reduced, so knowledge about how load transfer behaves can provide a good approach for estimating the potential grip. For this reason, the estimation of vertical load and slip angle can provide significant vehicle dynamic behaviour information.

The experiments have been carried out by means of an indoor tire test rig and strain sensors. Past researches in the field of intelligent tires indicated that strain sensors could meet the requirements to achieve an advanced intelligent tire system by means of strain measurement [23,24,25]. Moreover, strain sensors are less expensive than other types of sensors to measure strain data which are associated with tire behaviour and the tire-road contact patch. The strain gauges’ low cost as well as their robustness have been demonstrated to be suitable for developing intelligent tire applications.

## 2. Equipment and Methodology 

### 2.1. Strain-Based Test Equipment

**Figure 2:**
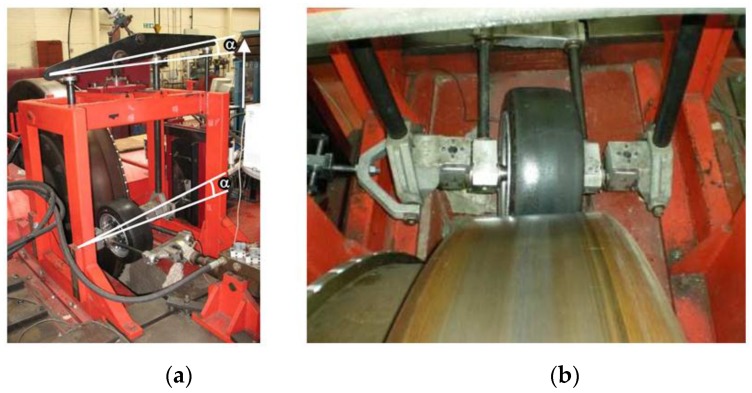
(**a**) Indoor tire test rig with the device to apply the slip angle; (**b**) Tire working in cornering conditions.

The test facility used for the experiments is an indoor tire test rig, which allows the speed, vertical load and slip angle to be varied. The strain-based experiment setup (including the indoor tire test rig equipped with the tire prototype) is shown in Figure 2a. The drum’s curved surface has an insignificant effect on the results because of its large diameter (2.44 m) which ensures that there is only a slight difference between a flat road and the large drum for tire/road contact. The error in contact length is less than 0.1% [26].

The test rig allows the lateral force to be measured when the slip angle is different from 0°, as shown in Figure 2b. It is the force that the tire transmits to the rolling contact surface in the lateral direction. Some previous works which focused on the effect of the lateral force have confirmed that it is directly associated to the slip angle and the potential adherence [11,27]. Overall, these studies indicated that the lateral and longitudinal tire-road forces depend on many factors such as the tire properties, the vertical load and other working conditions. As a consequence, the relation between them and the lateral force is quite complicated and nonlinear.

**Figure 3:**
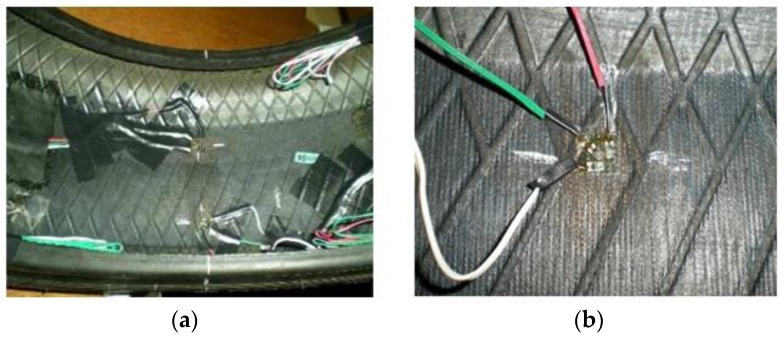
(**a**) Strain gauges’ setup; (**b**) Multiaxial gauge example.

Regarding strain sensors, some strain-based intelligent tire systems have been previously suggested in the literature. Some relevant researches measured strains on the tire tread, concluding that a strain sensor installed on either side of the centre line of the inner liner of the tire tread can provide useful information about the forces generated at the tire/road contact patch [23,24,25]. The strain sensors used in this study were located on either sides of the inner liner surface of the tire tread, as shown in Figure 3a. In this way, it is possible to measure and characterize loads and strains. However, measurements could be attenuated because of the tread thickness. For this reason, a data acquisition system with high resolution is especially necessary. In this paper, the accuracy as well as the robustness of the strain gauges were validated by tire tests carried out on the drum’s curved surface. Specifically, the tests have been carried out for over 50 h and the obtained data have shown very high repeatability. Figure 3b illustrates the support installation of the rectangular rosette strain sensors. It was made using the manufacturer recommended adhesive for rubber materials, so it can be assumed that the stiffness of the strain gauge doesn´t cause local stiffening effects. The strain gauge´s length is 2 mm with a gauge resistance of 120 Ω, being the resolution 0.001 με.

**Figure 4:**
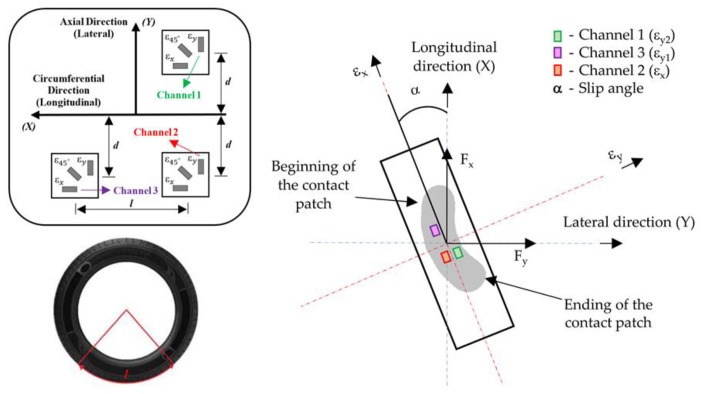
Strain gauges’ setup.

These strain gauges, which are applicable for large strain measurements, were attached at different points of the inner liner of the tire tread in order to measure lateral (ε_y2_—channel channel 1 and ε_y1_—channel channel 3) and longitudinal (ε_x_—channel 2) deformation, as shown in Figure 4.

It is important to show the strain gauges’ position. There are three multiaxial strain gauges, two of them in the same cross section and the third one separated by 123.75° of angular rotation. The distances “d” and “l” are about 0.040 m and 0.515 m, respectively. One of the major hindrances for developing an intelligent tire prototype is how to pack and route the wires to the outside of the tire. Despite the fact that in Figure 4 several channels are shown, not all of them were analysed in depth for two main reasons: some wires were damaged when passing them through the valves and others showed high similarity in the measurements and do not seem to provide any additional interesting information.

**Figure 5:**
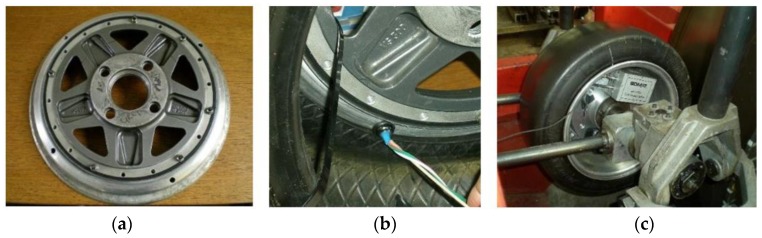
(**a**) Valves installed on the rim; (**b**) wires passed through the rim; (**c**) SoMat^®^ 2000 installed in the wheel.

In this work, three new valves were installed on the rim, as shown in Figure 5a,b. They were sealed with adhesive to ensure that the air didn´t leak through the valves’ holes and the tire was inflated to the desired inflation pressure. After that, the seal reliability was checked by leaving the tire for 72 h. These three new valves allow the connection between strain sensors and data acquisition system. In this work, a SoMat^®^ 2000 Field Computer (Somat Corporation, Urbana, IL, USA) was used as a data acquisition system, which is specially designed for portable data collection. The hardware of the system consists of a processor module, which includes the microprocessor, and a power/communication module, equipped with batteries, as shown in Figure 5c.

The SoMat^®^ 2000 has one Wheatstone strain bridge for each strain sensor, which can also be used in a quarter, half or full bridge configuration. The SoMat^®^ 2000 was driven by Test Control Software for Windows (WinTCS). When a test starts, the data acquisition module (i.e., the strain gauges module) is connected to the strain sensors. Secondly, the user can download the test configuration, designed using WinTCS, from a computer to the SoMat^®^ 2000 to initialize and run it. By sampling the electrical analogue signals, the data acquisition system can store and manipulate digital data as bytes of binary digits. In addition, it can provide a sampling frequency from 0.0005 to 5000 Hz, which ensures that the test strain data resolution is adequate to monitor enough strain points per wheel revolution. In this study, the sampling frequency was set to be 1000 Hz.

Additionally, different factors can affect the SoMat^®^ 2000 performance. They are usually divided into two categories: properties of the data acquisition system and the tire properties (such as tire radius or rolling speed). One of the critical point of the data acquisition system is the amount of collected data and the sampling frequency. It is obvious that a large number of data channels would increase the volume of the collected data, and as a consequence, a larger memory space would be required and the available test time would decrease (for a fixed memory size). The working range of the SoMat^®^ 2000 strain gage module used covered from −5000 to 5000 με.

The experiments consisted of studying the tread’s dynamic behaviour by changing the test conditions in order to determine the influence of some tire working conditions. The aforementioned investigation can be done by analyzing the measured strain sensor data, which were downloaded to a computer after the tests. For the experiments, a slick DUNLOP SP SPORT 175/505 R13 (tubeless) radial tire was used. This type of tire, which usually works under low vertical loads and inflation pressures, is used for the Formula SAE competition. The speed used in this work was limited at 50 km/h, which is suitable for the sampling frequency. In addition, the average speed in the circuits of the Formula SAE is usually under that speed.

### 2.2. Test Conditions

The operational range of parameters used for the cornering rolling conditions are:

- Tire inflation pressure: 0.8 bar–1.4 bar;
- Tire preload: 250 N–1000 N;
- Tire speed: 10 km/h–50 km/h;
- Tire slip angle: 0–14 degrees.

It is noteworthy that although different tests for slip angles between 0° and 14° have been carried out, we decided to not include data for 12° due to fact that the data acquisition system’s potentiometer did not provide a stable signal for that slip angle. This may indicate the occurrence of an adherence-slipping transition around 12° angle. This fact will be investigated further in future studies. Instead, it was decided to conduct trials at 13° instead, since between 0° and 10° trials have been performed at 2 degree intervals. Finally, it should be highlighted that the effect of temperature changes on the tire or the inflation gas are not taken into account in this work because the test conditions have been kept constant in terms of temperature and humidity (23 °C and 50% HR), following the recommendations of the strain gauge manufacturer.

## 3. Results

### 3.1. Experimental Data Analysis

The experimental data analysis allows us to extract some of the dynamic strain characteristics and deduce relations between strain data and tire working conditions. Despite the fact that some properties can be measured by different sensors, it must be asserted that the strain-based intelligent tire system proposed herein has the ultimate goal of providing a platform for reliable and accurate measurement or estimation of more tire operating parameters/variables using fewer sensors.

The variation in the strain data obtained could also be useful information to identify the variation of vertical load, rolling speed, etc. Since these strains are generated due to the corresponding working conditions, especially those which occur in the tire-road contact patch, their characteristics have a significant role in the estimation of tire dynamic behaviour and other characteristics [28].

This section is divided into two individual studies, each of which presents the analysis relating to each direction (i.e., lateral (ε_y_—channel channels 1 and 3) and longitudinal (ε_x_—channel channel 2)). Note that, channels 2 and 3 are placed in the outer part of the contact patch while the channel 1 is in the inner part when slip angle is different from 0° (see Figure 4). Before starting these studies, representative data curves were obtained taking into account the average strain for each point of the tire perimeter by averaging the separate cycles, which were examined under identical driving conditions.

Regarding the first study, the data analysis from channel 2 was of crucial importance in order to establish relations between longitudinal and lateral strain data in further studies. In the case of lateral direction, strain data from channels 1 and 3 have been collected. Since they are placed at the same distance from the center line of the inner tread tire, their behaviour when the working conditions change is quite similar.

**Figure 6:**
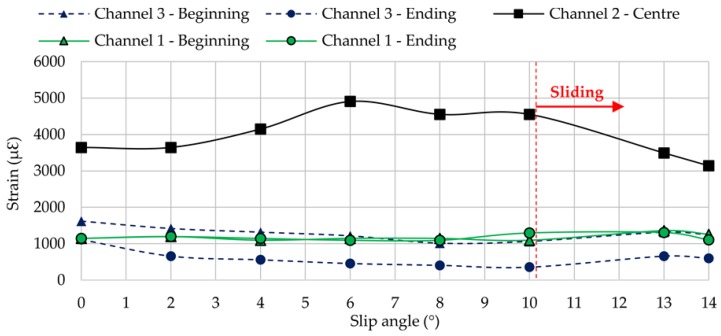
Maximum tension peaks in all channels (0.8 bar, 750 N, 30 km/h).

As it will be shown in the following sections, each channel shows some representative peaks in the strain curves. Channel 2 (longitudinal direction) exhibits a maximum tension peak at the center of the contact patch while channels 1 and 3 (lateral direction) exhibit two maximum tension peaks, one at the beginning of the contact patch and another at the ending. The variation of these maximum strain peaks is showed in Figure 6 at 0.8 bar, 750 N and 30 km/h.

**Figure 7:**
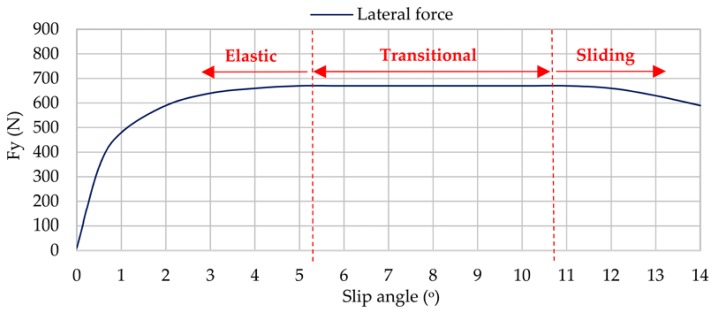
Lateral force measured by the test rig (0.8 bar, 750 N, 30 km/h).

On the other hand, as was mentioned in the previous section, the indoor test rig allows us to obtain information about the lateral force. Figure 7 shows data provided by the indoor test rig, which represent clearly how lateral force changes when slip angle increases.

When cornering, car tires deform laterally and longitudinally, causing changes in the tire tread deformation. As a consequence, tires sustain lateral forces in order to drive the vehicle along a certain path. If the road is capable of absorbing these forces, the tire does not slide and, as a result, the vehicle follows the desired path. The relation between lateral forces and slip angles determines the vehicle´s lateral dynamics.

The curve of lateral force vs. slip angle is commonly divided into three regions: linear/elastic, transitional, and sliding [29]. Taking a look at Figure 7, it is significant that between 5° and 11° (i.e., the transitional region) the lateral force reaches their maximum values, which means the tire is working at the limit of adhesion. Although from 0° to 5° and from 11° to 14° optimum conditions are not reached, it could be due to different reasons. Between 0° and 5° (i.e., elastic region) the tire is not subjected to excessively demanding conditions, so force transmitted to the indoor test rig is lower than the maximum value that could be transmitted. However, from 11° (i.e., sliding region), the type of surface begins to affect the operation of the tire, that is, the contact surface is not able to transmit the force that the tire needs in order not to slide on the surface.

Figure 6 and Figure 7 reveal that strain data and lateral force are closely related. When tire starts sliding (from 10° approximately, Figure 7), the maximum strain peaks in all channels change significantly, especially in channels 2 and 3 (see Figure 6). In addition, contrary to channel 1, channel 3 shows significant differences between maximum tensile strain peaks at the beginning and ending of the contact patch (see Figure 6). For these reasons, strain curves in lateral direction are shown for channel 3 (ε_y1_), since it is presumably more useful for the development of the intelligent tire, while in longitudinal direction strain curves are shown for channel 2 (ε_x_). Finally, although the relationship between strain data and lateral force will not be discussed in this paper, it is clear this is a line of research worthy of in-depth study soon.

### 3.2. Strain Data Changes in Longitudinal Direction

**Figure 8:**
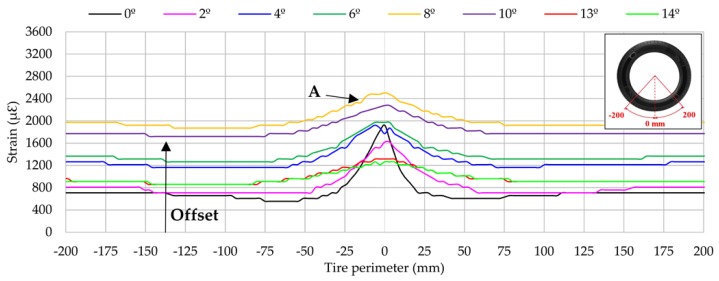
Influence of slip angle (channel 2 at 1.2 bar, 250 N and 10 km/h).

This section studies closely the influence of the slip angle in longitudinal direction around the centre of contact patch. Data from channel 2 are presented in Figure 8 for the interval (−200, 200) mm of the entire tire perimeter (1592.6 mm).

Peak strain values increase gradually when the slip angle increases from 2° to 10°. However, at 13° and 14° strain these values decrease dramatically, even below strain data at 0°. It seems to be related to the loss of adherence that occurs in Figure 8 from 11°. Besides, the curve shape is progressively less pronounced around the center of contact patch (A) when slip angle increases. That is, for slip angles near to 13°, the tire starts sliding. As a result, the adherence of the tire tread decreases as well as strain data around the contact patch. For instance, at 13° the maximum tension peak decreases by almost −32% and at 14° by −34% in comparison with the same peak at 0°.

In addition, apart from the information provided by the peak at A, it should be highlighted that the data also provides useful information when the strain sensor is distant to the centre of contact patch. For instance, at −150 mm (see Figure 8), it is observed that the offset value changes depending on some working conditions. In particular, it is observed that the offset value increases as slip angle increases. However, note that, offset values at 13° and 14° tend to decrease in the same way that happened at peak A.

**Figure 9:**
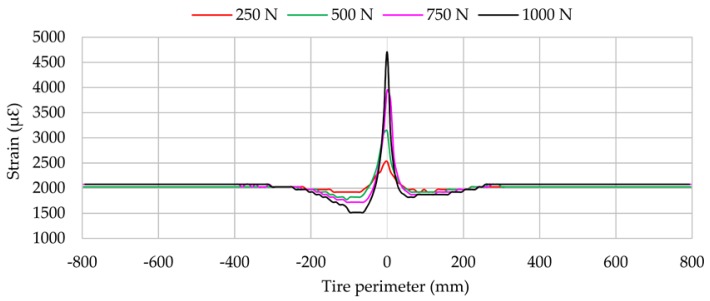
Influence of vertical load (channel 2 at 1.2 bar, 30 km/h, 8°).

Vertical load is the most influential parameter on longitudinal strain (ε_x_), as shown in Figure 9. Changes in peak strain at the centre of contact parch are practically proportional to the vertical load change. In comparison with the maximum strain peak at 250 N, maximum strain increases by 24% at 500 N, by 56% at 750 N and by 86% at 1000 N.

**Figure 10:**
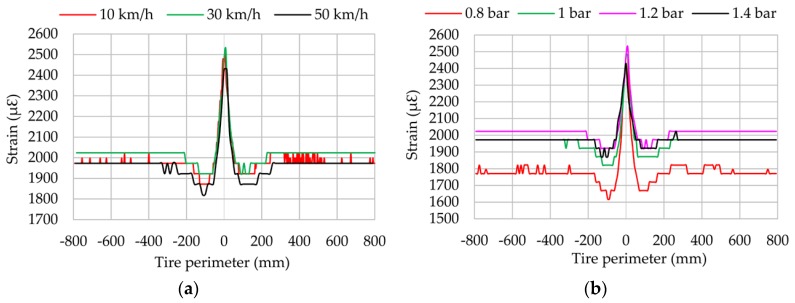
(**a**) Influence of speed; (**b**) Influence of pressure (channel 2 at 1.2 bar, 250 N, 8°).

On the other hand, the vertical load increment has more influence at the beginning of the contact patch (close to −100 mm), where the maximum compressive strain value appears, than at the ending, as shown in Figure 9. Since channel 2 (see Figure 9) measures in longitudinal direction, which is directly related to the processes of acceleration and braking, the differences between the maximum values of compressive strain at the beginning and ending of the contact patch may be due to cornering conditions in these parts of the contact patch are involved in acceleration and braking processes. This seems to show that the tread parts on different sides of tread centre line are working in different conditions, so the cornering behaviour is caused by one side being in traction and the other being in braking. This hypothesis will be analysed in-depth in further studies. Finally, note that vertical load has hardly any influence on the offset value. Regarding the influence of rolling speed and inflation pressure, Figure 10a,b show that, even though they affect the maximum strain values, this influence is not always linear.

Figure 10b illustrates that the strain curve amplitude (i.e, the difference between maximum tensile and compressive peaks) is higher for 0.8 bar than for the other inflation pressure values. Otherwise, the amplitude values for 1, 1.2 and 1.4 bar remain practically constant, with differences under 10% between them. Additionally, the offset value for 0.8 bar is clearly lower than in the other cases. All this seems to show that for inflation pressures over 1 bar, the stiffness is higher and limits the differences in terms of amplitude of the strain curves. On the other hand, when the tire is underinflated, the strain curve amplitude increases and the offset value decreases.

### 3.3. Strain Data Changes in Lateral Direction

**Figure 11:**
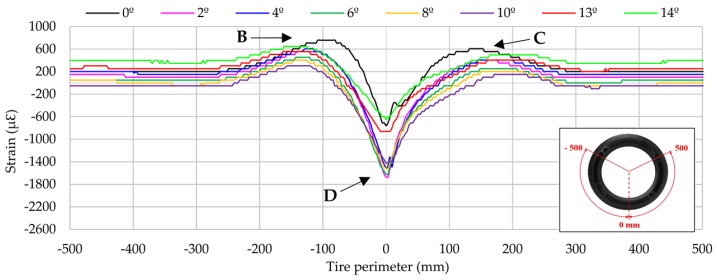
Influence of slip angle (channel 3 at 1.2 bar, 250 N, 10 km/h).

As mentioned previously, in the lateral direction the most useful information is provided by channel 3 (ε_y1_), which is placed on the inner part of the contact patch. Variations on the strain data when the slip angle changes are very significant, as shown in Figure 11. In this figure, strain data in the interval (-500, 500) mm of the entire tire perimeter are shown. As it has already been mentioned, for the interval between 6° and 10°, the limit of adhesion for this type of tire in the indoor test rig surface occurs (see Figure 7a).

Regarding the peak strain values, they change slightly between the different slip angles in tensile strain peaks at the beginning (B) and ending (C) of the contact patch. Up to 8° tensile strain values increase as the slip angle increases, nevertheless, at 10° the strain peak is lower at the beginning than at the ending of the contact patch. The most significant change in trend occurs in compressive strain peak values at D. They change continuously up to 10°, but then decrease dramatically at 13° and 14°. This fact suggests that the variation of the maximum compressive strain values could be directly related to tire grip.

In the same way, as in the longitudinal direction, the offset value changes depending on the working condition. In Figure 11 the offset values tend to decrease to 0 με when sliding is happening (from 13°). Thus, despite the fact that the peaks of the curves have been highlighted as the most interesting in order to identify them in real-time, the information provided by the offset value could also be interesting for further studies.

It is also interesting to mention that the increment in the slip angle causes an increment in the contact patch length, given that the strain variations extend further away from the centre of contact patch (0 mm).

**Figure 12:**
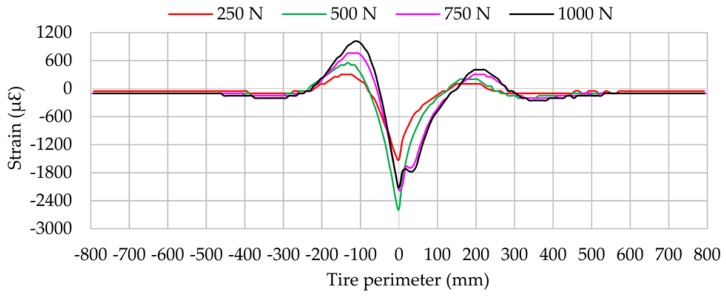
Influence of vertical load (channel 1 at 1.2 bar, 30 km/h, 8°).

In the same manner as in the longitudinal direction, vertical load changes affect notably the strain values, especially at the beginning and ending of the contact patch, as shown in Figure 12. Peak tensile strain increases linearly at the beginning and ending of the contact patch when vertical load increases. However, maximum compressive strain values are not linear. It thus indicates that the most useful information may be in the maximum tension values, especially at the beginning of the contact patch.

**Figure 13:**
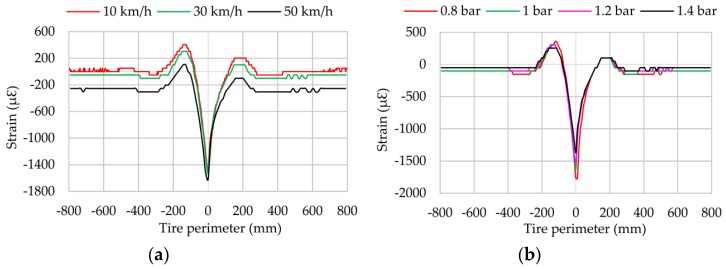
(**a**) Influence of speed; (**b**) Influence of pressure (channel 3 at 1.2 bar, 250 N, 8°).

Regarding the influence of speed, Figure 13a shows that it affects the maximum tensile strain values and seems to suggest that the effect of the speed is simply to shift the curve upward. This could be caused by the effect of centrifugal force, but this aspect should be analysed deeply in further works.

Although the maximum compression peaks are almost the same at 10 and 30 km/h, it can be assumed that the higher the speed, the more pronounced maximum compression peak appears. Figure 13b displays the influence of pressure on strain data when slip angle is different from 0°. Contrary to longitudinal direction, strain data decreases in lateral direction as inflation pressure increases due to the increase in tire stiffness. This effect is clearly shown in the maximum compression values. Strain data at the beginning and ending of the contact patch are not clear enough, in contrast with the influence of vertical load.

## 4. Fuzzy Logic Implementation

In this section, the fuzzy logic computational method used to estimate slip angle and vertical load is described. As it is well known, when the load transfer is increased, the effective adherence is lower. In addition, as it has been mentioned, the slip angle contributes hugely to subjecting the tire to more demanding conditions, which could result in the loss of adherence, so the knowledge about the effect of load transfer and slip angle may provide a good approach to known approximately the potential adherence. Simulation results demonstrate the potential and feasibility of strain sensors to estimate tire working conditions by measuring tire dynamic strain characteristics. Finally, a Sliding Detection System (SDS) methodology using fuzzy logic and strain data is proposed in the last part.

### 4.1. Fuzzy Logic Framework

Fuzzy logic is a method that allows us to solve nonlinear systems by estimating parameters providing some initial values. The fuzzy logic system has been developed using MATLAB/Simulink® (The Math-Works, Natick, MA, USA). A brief overview of the generic framework of the fuzzy-logic inference system is given in this section.

**Figure 14:**
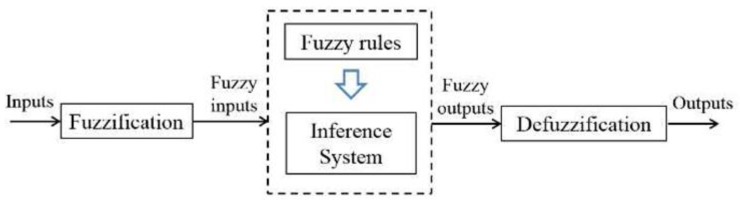
Scheme of a fuzzy system.

The procedure in which the Inference System deduces the output values based on inputs is usually described in three steps (see Figure 14): fuzzification, fuzzy inference and deffuzification. More information of a fuzzy set can be found in [30].

**Figure 15:**
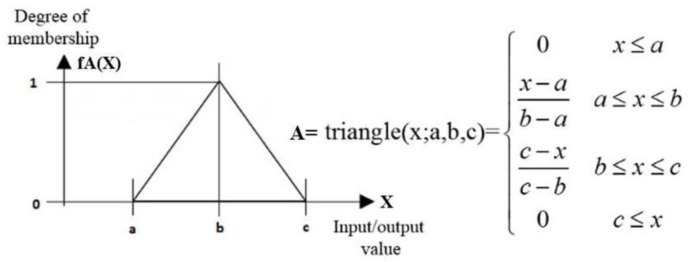
The membership functions for a generic variable.

The design of a fuzzy logic system requires a set of membership functions for inputs and outputs. As in [25], the Mamdani´s method as well as triangular membership functions are used. Figure 15 represents the mathematical expressions of the functions. Note that, the higher value of fA(X), the higher degree of belonging to the corresponding triangle is obtained.

After defining the membership functions, the fuzzy rules work together to estimate the most probable output taking into account the known relationships [25,30].

### 4.2. Slip Angle and Vertical Load Estimation

In this particular work, a fuzzy logic system to estimate the slip angle and the vertical load has been developed. To do this, some significant points of experimental strain curves have been used as the inputs of the developed fuzzy logic.

**Figure 16:**
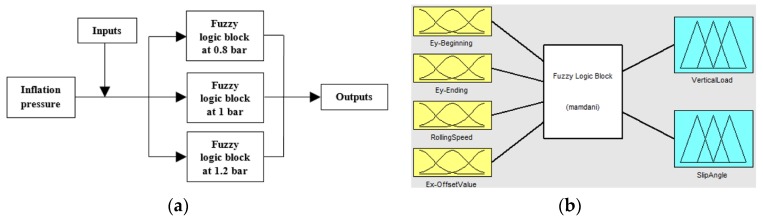
(**a**) Working scheme used to obtain outputs; (**b**) Fuzzy Logic toolbar interface.

Since the rolling speed can be accurately estimated by the period from peak to peak in strain curves, it has been taken as an input parameter. Besides, although the inflation pressure could be estimated from tire’s deformation, it can be correctly measured by current systems, for this reason, in this simulation work it has been assumed as a known parameter. In this way, three fuzzy logic blocks have been developed for 0.8, 1 and 1.2 bar, since 1 bar is considered the ideal pressure of the tires used in the Formula SAE competition. Thus, once the inflation pressure is known, the corresponding fuzzy logic block is used (see Figure 16a).

To sum up, three inputs from strain curves have been considered: the maximum tensile strain peaks from channel 3 at the beginning and ending of the contact patch (points B and C, see Figure 11) and the offset values from channel 2 (see Figure 8). Since the offset value only changes significantly with variations of slip angle in comparison with other parameters, it has been also considered as a known parameter in order to get better estimations of the slip angle. For better understanding, Figure 16b represents in a simplified form the fuzzy logic toolbox interface besides the inputs and outputs considered.

As can be seen, the fuzzy logic rules that define the relationship between inputs and outputs are crucial to develop the fuzzy logic system, since they indicate the influence of vertical load and slip angle in tire´s deformation.

**Figure 17:**
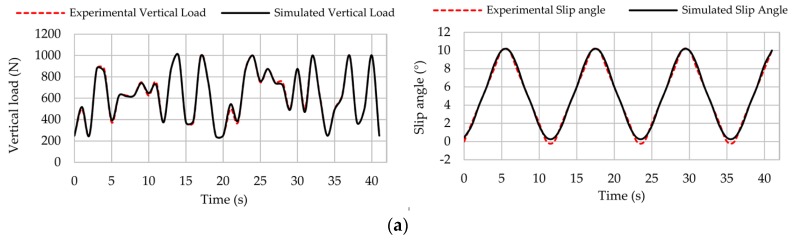

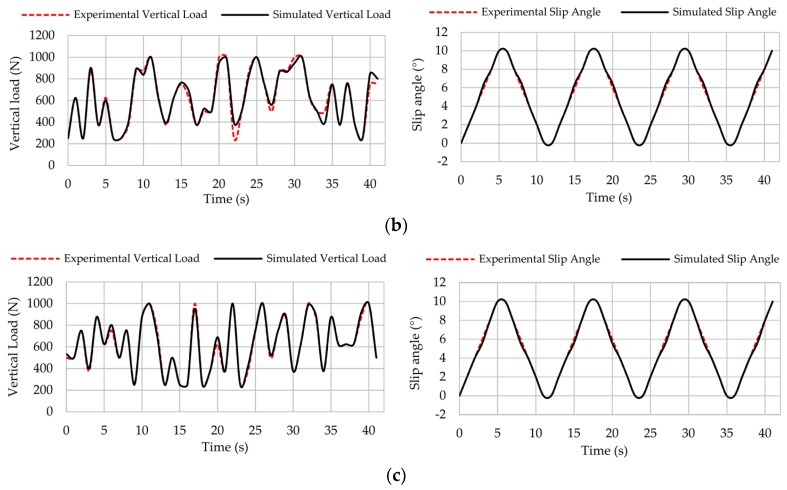
Simulation results for vertical load (left) and slip angle (right) at: (**a**) 10 km/h, 0.8 bar; (**b**) 30 km/h, 1 bar; (**c**) 50 km/h, 1.2 bar.

Figure 17a–c show some simulation results for vertical load and slip angle. These simulations have been carried out at 10, 30 and 50 km/h (under steady conditions) and different pressures (0.8, 1 and 1.2 bar) assuming that the driver turns the steering wheel from one side to another in a way that the slip angle varies from 0° to 10° and vice versa.

Figure 17 shows that simulation results are consistent for all cases, since experimental and simulation curves are practically overlapped. However, it should be pointed out that better results could be obtained using a more complete data set. In addition, despite the fact that the output parameters do not show high consistency, it does not imply an obstacle, since they can independently change if the rolling surface has banks, potholes, or the tire is involved in acceleration or braking processes.

Together with tire acceleration and the vehicle characteristics, the estimated parameters can be significantly useful to estimate many other parameters of the vehicle dynamic behaviour such as the friction coefficient or load transfer. Although they are a work to be carried out, estimation results are promising regarding the potential grip estimation, which is the ultimate goal of intelligent tires. Table 1 lists the average errors for the simulation results.

As it can be observed, the average error is lower than 6% for any simulation condition. In total, the average error for the vertical load and slip angle results are 3.08% and 2.47%, respectively. As Table 1 shows, estimations of vertical load and slip angle are not as good as the others at 0.8 bar and 50 km/h. It is because the obtained average value of the offset measurement is very similar to higher slip angle offset values, as explained in the next section. Therefore, estimations in this case need to be improved. Overall, the fuzzy logic provides better estimations of the slip angle because the offset values, which have been used to estimate it, depends mainly on rolling speed and slip angle. On the contrary, the maximum tensile strain peaks used to estimate vertical load (points B and C, see Figure 11) depend on all the parameters changed during the experiments. Thus, it is demonstrated that the proposed method can accurately estimate the slip angle and the vertical load from strain data.

### 4.3. Sliding Detection System (SDS) Proposal

The capability of tires to estimate the potential friction has always been the final goal of intelligent tires studies. However, it should not be forgotten that its estimation must be useful to other active control systems and contribute as an active safety system. In this sense, a Sliding Detection System (SDS) using strain data and the developed fuzzy logic is suggested herein.

**Figure 18:**
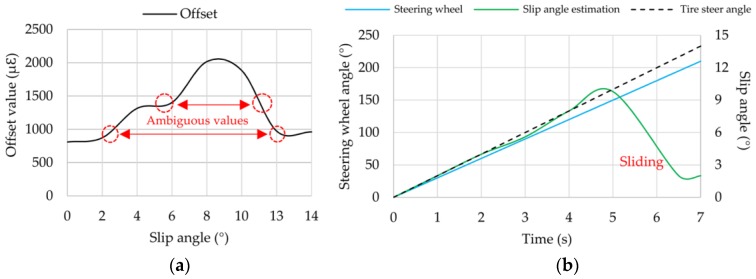
(**a**) Ambiguous values in offset data; (**b**) Example of SDS at 1.2 bar, 250 N, 10 km/h.

Despite the fact that the range covered by the experiments comprise slip angles from 0° to 14°, simulation results were shown only up to 10°. As shown in Figure 18a, because strain data reduce drastically when the tire starts sliding (slip angles over 10°), offset values for 13° and 14° give similar results as in case of 0°, 2° or 4°. Thus, the offset measurement that serves mainly to estimate the slip angle has not only one value for each slip angle. Contrary to what it might seem, this ambiguity may be used to develop a sliding detection system (SDS). As a way of example, let’s assume that the steering wheel and the tire steer angle are monitored while driving (for instance, taking a 15:1 relation between them and using a steering angle sensor to measure the steering wheel angle). Then, if steering wheel angle is increasing and the estimated slip angle (by means of collected strain data in real-time) is decreasing, the SDS could alert the driver that the tire is losing adherence before an accident occurs. Furthermore, this effect depends also on the load transfer in heading processes and should be considered.

Therefore, strain sensors, in combination with estimation methodologies, have the potential to serve as an active safety system for drivers and control systems. If sliding conditions are detected, the tire could provide this information to ABS or ESC to take action without loss of the vehicle control. In this way, despite the fact that this SDS is just a suggestion to be developed in the near future, it could contribute greatly to accident prevention and thus enhance the tire’s role in improving vehicle safety.

## 5. Discussion

As in previous studies, a major aspect of the work presented in this paper is to investigate the feasibility, robustness and prospects of a strain-based intelligent tire to estimate tire working conditions and provide information to other vehicle control systems.

Results have shown that strain gauges can measure accurately the tire behaviour in cornering conditions under steady state, without getting the tire involved in severe acceleration or braking processes. The obtained results have confirmed that the shape of the strain response curve is considerably different depending on the working conditions. When the slip angle increases, it clearly affects the strain data during the compression process in lateral direction and during the tension process in longitudinal direction.

Longitudinally (see Figure 8), maximum tensile values (A) and offset values increase as the slip angle increases from 2°, except at 13° and 14°. This fact is particularly interesting due to the expectation that the lateral force decreases from 13°. This phenomenon also occurs in lateral direction, but to a lesser extent than in the longitudinal direction.

Furthermore, as in longitudinal direction there is only one significant point to consider, sensors arranged longitudinally are particularly interesting to develop the intelligent tire, because the identification of the maximum strain value and offset value would be easier. Regarding the lateral direction (see Figure 11), at point D the strain variation is especially pronounced when the tire starts sliding, decreasing dramatically from 13°.

Results for vertical load changes are practically linear in both the lateral direction and the longitudinal direction (see Figure 9 and Figure 12). When the vertical load increases, the maximum tensile or compressive strain peaks also increase. However, regarding inflation pressure and speed changes, deformation data provided by lateral strain gauges are recommended. Data measured by longitudinal strain gauges are not linear, and as a consequence, maximum tension and compression points are not as easy to recognize as in the case of lateral strain data.

Finally, simulations by means of fuzzy logic have demonstrated that strain sensors in combination with computational methods are feasible to develop the intelligent tire system. The computational method used herein is capable of solving the non-linearity characteristics of the tires’ parameters and turn tires into a source of information. However, in order to afford scenarios under longitudinal and lateral slip combined conditions, the proposed method should be optimized [31]. In addition, the possibility of using other estimation method will be assessed in the near future [32].

## 6. Conclusions

In this paper, tests under different tire working conditions during cornering have been performed. The main equipment used has been a data acquisition system, indoor test rig, a tire and strain sensors. The obtained data has been used to estimate vertical load and slip angle.

The intelligent tire prototype used in this study has also shown that strain sensors are suitable for measuring tire deformation, mainly maximum values of tensile and compressive strain. These peak values can be used to estimate potential grip as well as working conditions, since when sliding begins, peak strain values in the inner liner of the contact patch decrease drastically. However, taking into account the computation time demands, the developed estimation method must be optimized to be capable of estimating conditions in real-time. In addition, it is demonstrated that the shape of the strain response curves can provide useful information regarding working conditions and even if the tire is subjected to cornering conditions, where requirements are greater.

Future work will be focused on estimating the potential grip and detecting the tire’s sliding in real-time by measuring the tire’s deformation. Secondly, a detailed study of maximum tensile and compressive strain values for higher speeds is needed. Another possible future research area would be to install sheets with different roughness on the drum surface, which would be necessary to determine how strain data change for different working conditions and different surfaces. Finally, performing test with different types of tires and the interaction between measured data and vehicle dynamic control systems such as ABS or ESC is also an objective to be achieved.

## Figures and Tables

**Table 1:** Average error of vertical load and slip angle simulation results.

Pressure (bar)	Speed (km/h)	Vertical Load Error (%)	Slip Angle Error (%)
0.8	10	1.36	3.71
	30	1.64	0.314
	50	5.42	11.29
1	10	3.80	1.82
	30	4.18	1.52
	50	3.81	0.78
1.2	10	3.64	0.73
	30	2.28	0.69
	50	1.63	1.38
